# Structure–function analyses of coiled-coil immune receptors define a hydrophobic module for improving plant virus resistance

**DOI:** 10.1093/jxb/erac477

**Published:** 2022-12-06

**Authors:** Xiujuan Wu, Xuan Zhang, Hongwei Wang, Rong-xiang Fang, Jian Ye

**Affiliations:** State Key Laboratory of Plant Genomics, Institute of Microbiology, Chinese Academy of Sciences, Beijing 100101, China; CAS Center for Excellence in Biotic Interactions, University of Chinese Academy of Sciences, Beijing 100049, China; State Key Laboratory of Plant Genomics, Institute of Microbiology, Chinese Academy of Sciences, Beijing 100101, China; State Key Laboratory of Plant Genomics, Institute of Microbiology, Chinese Academy of Sciences, Beijing 100101, China; CAS Center for Excellence in Biotic Interactions, University of Chinese Academy of Sciences, Beijing 100049, China; State Key Laboratory of Plant Genomics, Institute of Microbiology, Chinese Academy of Sciences, Beijing 100101, China; CAS Center for Excellence in Biotic Interactions, University of Chinese Academy of Sciences, Beijing 100049, China; State Key Laboratory of Plant Genomics, Institute of Microbiology, Chinese Academy of Sciences, Beijing 100101, China; CAS Center for Excellence in Biotic Interactions, University of Chinese Academy of Sciences, Beijing 100049, China; Bielefeld University, Germany

**Keywords:** Coiled-coil domain, hydrophobic groove, NLR, Tsw, TSWV, virus resistance

## Abstract

Plant immunity relies on nucleotide-binding oligomerization domain (NOD)-like receptors (NLRs) that detect microbial patterns released by pathogens, and activate localized cell death to prevent the spread of pathogens. *Tsw* is the only identified resistance (*R*) gene encoding an NLR, conferring resistance to tomato spotted wilt orthotospovirus (TSWV) in pepper species (*Capsicum*, *Solanaceae*). However, molecular and cellular mechanisms of *Tsw*-mediated resistance are still elusive. Here, we analysed the structural and cellular functional features of Tsw protein, and defined a hydrophobic module to improve NLR-mediated virus resistance. The plasma membrane associated N-terminal 137 amino acid in the coiled-coil (CC) domain of Tsw is the minimum fragment sufficient to trigger cell death in *Nicotiana benthamiana* plants. Transient and transgenic expression assays in plants indicated that the amino acids of the hydrophobic groove (134^th^–137^th^ amino acid) in the CC domain is critical for its full function and can be modified for enhanced disease resistance. Based on the structural features of Tsw, a super-hydrophobic funnel-like mutant, Tsw^Y137W^, was identified to confer higher resistance to TSWV in a *SGT1* (Suppressor of G-two allele of *Skp1*)-dependent manner. The same point mutation in a tomato Tsw-like NLR protein also improved resistance to pathogens, suggesting a feasible way of structure-assisted improvement of NLRs.

## Teaser

The 137-amino acid plasma membrane-associated coiled-coil domain of Tsw NLR protein from pepper functions in *Tsw*-mediated resistance.

## Introduction

Lack of robust genetic resistance to infectious microorganisms in crops is one of the major reasons for the rapid spread of many microbial diseases that severely reduce crop yield and quality annually. We have enough understanding of the genetic networks controlling plant immune systems now, and this knowledge can be applied to engineer pathogen-resistant plants. Plants employ multiple layers of defence, including disease resistance (*R*) genes, RNA silencing, and defensive phytohormone signalling pathways, to prevent the spread of pathogenic diseases and plant death (Zhou and Zhang, 2020; Ye *et al.*, 2021). *R* genes typically encode nucleotide-binding leucine-rich repeat (NLR) proteins on plant cell surfaces or intracellular regions to perceive microbial pathogen-derived molecules (effectors), executing immune signalling by initiating the activation of programmed cell death (Tang *et al.*, 2017; Kourelis and van der Hoorn, 2018; Van de Weyer *et al.*, 2019; Zhou and Zhang, 2020).

The recent integration of the combined knowledge generated from structural, cellular, and biochemical analyses of NLR-mediated plant immunity has demonstrated that both the indirect and direct recognition of pathogens can trigger the oligomerization of plant NLRs into active complexes. Recent findings on *Arabidopsis thaliana* ZAR1 (HOPZ-ACTIVATED RESISTANCE1) have demonstrated that the activated resistosome could result in an array of immune signals including the influx of calcium (Ca^2+^), which triggers immune responses that often culminate in host cell death to block pathogen survival (Wang *et al.*, 2019a, Ma *et al.*, 2020, Bi *et al*., 2021). In particular, the amino acid situated in the channel pore of N-terminal ZAR1 CC (coiled-coil) domain determines its ion channel activity. In fact, the CC domain of NLRs is usually considered as the signalling execution domain of NLRs; isolated CC domains of several NLRs are reported to induce cell death (Horsefield *et al.*, 2019; Baudin *et al.*, 2020). Moreover, the CC domain is also associated with the indirect surveillance of pathogen effectors, and could directly bind downstream signalling molecules (Kourelis and van der Hoorn, 2018). These modular architectures of plant NLRs offer a flexible platform for developing diverse strategies to perceive pathogen effectors, and to enhance the strength of the immune response. A pioneering study has shown that the protease cleavage site of PBS1 (*Avrpphb susceptible1*), which activates the NLR protein RPS5 (Resistance to *Pseudomonas syringae* 5) when cleaved, can be genetically modified to detect protease effectors from a wide range of pathogens. This strategy is expected to be effective to expand the pathogen recognition spectrum in the engineered plant (Kim *et al.*, 2016). Beside this conceptive strategy, we hypothesized that it might be possible to develop more effective immunity against pathogens by structure-function analysis, which could assist improvements of NLRs for crops lacking known disease resistance genes.

Tospoviruses (*Bunyaviridae*), mainly the tomato spotted wilt orthotospovirus (TSWV), represent a constant threat to plants, through infecting about 800 plant species from over 50 families, and causing substantial crop losses worldwide (Oliver and Whitfield, 2016). TSWV mainly transmits among plants through their insect vector thrips, and could also be seed transmissible (Wu *et al.*, 2019; Wang *et al.*, 2022). Natural genetic sources of germplasms and resistance to tospoviruses for crop breeding are very limited, and new breeding and biotechnological approaches to obtain resistant crop cultivars are in high demand. Several groups, including ours, have demonstrated that a major defence phytohormone jasmonate plays critical roles in resistance against replication of tospoviruses and viral trans-plant transmission (Abe *et al.*, 2008; Wu *et al.*, 2019). Beside phytohormone-mediated host defence, so far only two CC-type NLR (CNL) resistance proteins against tospoviruses have been identified (Boiteux, 1995; Brommonschenkel *et al.*, 2000; Spassova *et al.*, 2001; Kim *et al.*, 2017). Tomato Sw-5b is a CNL protein with one extra N-terminal domain, which coordinates with the CC domain to regulate its autoinhibition and activation (Chen *et al.*, 2016; Zhu *et al.*, 2019). The movement protein (NSm) of TSWV is recognized by tomato Sw-5b (De Oliveira *et al.*, 2016; Zhu *et al.*, 2017). Another CNL protein Tsw confers pepper resistance by detecting the effector protein Non-structural protein (NSs) of TSWV, but the induced resistance shows low efficacy, a narrow pathogen spectrum, and temperature sensitivity, and has been frequently overcome by rapidly evolving pathogens in the pepper *Capsicum* (Turina *et al.*, 2016; Kim *et al.*, 2017; Chung *et al.*, 2018; de Ronde *et al.*, 2019; Yoon *et al.*, 2021). Meanwhile, tospoviruses have evolved to suppress host defences to promote the population of the insect vector, thereby expanding the disease pandemic (Wu *et al.*, 2019; Wu and Ye, 2020). Considering that the economic impact of tospoviruses has much increased during the last three decades, it is necessary to reinforce the efforts to characterize new/alternative resistance genes, and search for improvements on known NLR-involved immunity against tospoviruses.

In this study, we functionally characterized Tsw as a CNL with a 137 aa plasma membrane-associated CC domain that acts as an executor for *R*-gene-mediated resistance signalling. More importantly, we identified a hydrophobic module in the CC domain for improving disease resistance with NLRs. We verified this strategy on two *Solanaceae* CNL proteins [pepper Tsw and its paralog in tomato (*Solanum lycopersicum*)] in another *Solanaceae* plant, *Nicotiana benthamiana*. A simultaneous substitution of Tyr^135^ or Tyr^137^ to tryptophan in the hydrophobic groove of NLRs improved the disease resistance efficacy both in *N. benthamiana* and pepper. This module of NLRs could be further exploited in disease resistance breeding programs via genome editing in the future.

## Materials and methods

### Plant materials and plasmids

*Nicotiana benthamiana* plants were grown in growth chambers at 23 °C with a 12 h light (80 μmol m^–2^ s^–1^)/12 h dark cycle. The transgenic *N. benthamiana* plants harbouring *Tsw* and *Tsw*^*Y137W*^ were generated by transforming them with pMDC32-Tsw-3xFlag and pMDC32-Tsw^Y137W^-3×Flag constructs, respectively. In detail, TswY137W was obtained by mutating the nucleotides TAT409–411 to TGG409–411 using mutant primers listed in Supplementary Table S1, through PCR. Subsequently, the amplified *Tsw* and *Tsw*^Y137W^ gene coding sequences were ligated to pMDC32 empty vector using the *Kpn*I and *Spe*I restriction sites. A widely used chilli pepper cultivar (*Capsicum annuum* L. ‘GuoFu 208’) in Northern China was used for the disease resistance assay. For constructing the expression vectors for the full length or domains of the NLRs, codon-optimized *Tsw* and tomato *Tsw* paralog coding sequences were artificially synthesized (GENEWIZ,USA) and cloned into the plant binary vector pGDGm, which contains the GFP tag on the C-terminus of genes (Goodin *et al.*, 2002; Curtis and Grossniklaus, 2003). Site-directed mutants were generated using specific primers carrying the desired mutations, as described previously (Li *et al*., 2014). The entry vector pENTR-3C in the Gateway system (Invitrogen, A10464, USA) and destination vectors pBA-DC-6Myc, pH7WG2Y under the control of a CaMV 35S promoter were used for *35S:CC-Myc* and *35S:YFP-CC* constructs. Primers used in this study are listed in Supplementary Table S1.

### Virus inoculation

The leaves of TSWV-infected *N. benthamiana* plants were kindly provided by Xiaorong Tao (Nanjing Agriculture University, China) initially and maintained in our laboratory by mechanical inoculation to *N. benthamiana.* These were used for the virus infection assay. Briefly, 0.1 g virus (isolate TSWV-YN) infected-leaves were ground to a powder in liquid nitrogen, and then dissolved in 5 ml of 0.05 M phosphate buffer (pH 7.0). Subsequently, 150 μl of virus containing supernatant was mechanically inoculated onto *N. benthamiana* leaves, as described previously (Wu *et al.*, 2019).

### Agroinfiltration, transient expression, and western blot analysis

All expression vectors were transformed into *Agrobacterium tumefaciens* strain EHA105*. Agrobacterium* carrying the binary vectors were cultivated overnight and diluted, then infiltrated into the abaxial sides of *N. benthamiana* leaves. The cell death symptoms were observed at 3 days (CC domain) or 7 days (full length NLR) post-inoculation (dpi) and photographed under UV/white light. To analyse protein expression, treated leaves were harvested and tested by immunoblotting, as described previously (Zhao *et al.*, 2019). Total proteins were extracted from infiltrated leaves, separated using 10% SDS-PAGE, and transferred to a polyvinylidene difluoride (PVDF) membrane (Merck Millipore, IPVH00010, Germany). Finally, pGDGm-CC series plasmids which encode the GFP epitope-tagged proteins, were detected using an anti-GFP monoclonal antibody with 1:5000 dilution ratio (TransGen Biotech, China).

For virus inoculation, *Agrobacterium* carrying the *Tsw*-expressing vectors was infiltrated into *N. benthamiana* or pepper leaves, followed by mechanical inoculation with TSWV at 2 dpi. Viral abundance in *N. benthamiana* or pepper plants was detected by RT–PCR and with antibodies against NSs and Ncp, at 7 dpi or 14 dpi, respectively.

### Confocal microscopy

*A. tumefaciens* containing the pGDGm vector with CC and CC mutant were transiently expressed in 6–8-week-old *N. benthamiana* using agroinfiltration. Protein sub-cellular localization was detected at 2 dpi. For plasmolysis assay, agroinfiltrated *N. benthamiana* leaves were treated with 5% NaCl for 5–10 min to trigger cell plasmolysis, before observation. Confocal imaging was observed and recorded with a Leica SP8 laser scanning confocal microscope (Leica, Germany).

### Membrane fractionation

Plasma membrane partitioning was performed with Minute^TM^ Plasma Membrane Protein Isolation Kit, according to the manufacturer’s protocol (Invent, SM-005-P, USA). The anti-H^+^-ATPase monoclonal antibody (Agrisera, Sweden) was used to detect the plasma membrane marker protein, H^+^-ATPase.

### Co-immunoprecipitation assay (Co-IP)

*A. tumefaciens* containing the pGDGm-CC and empty vector pGDGm plasmids were co-infiltrated with *A. tumefaciens* containing the *35S:CC-Myc* plasmids into *N. benthamiana* leaves individually. After 3 d, ~1 g of leaf tissue was collected and ground to a powder in liquid nitrogen, as described previously (Wu *et al.*, 2019). Total proteins were extracted in 1 ml of extraction buffer (25 mM Tris-HCl, pH 7.5, 150 mM NaCl, 1 mM EDTA, 0.5% NP-40, 10% glycerol, 10 mM DTT, one tablet of protease inhibitor cocktail per100 ml; Sigma-Aldrich, USA). Then the protein extracts were incubated with 25 μl of GFP-trap beads (Lablead, China, 5500041921) for 3 h at 4 °C. The beads were then washed three times with extraction buffer and resuspended in 2×SDS buffer before using for immunoblotting analysis.

### Blue native–polyacrylamide gel electrophoresis (BN-PAGE)

BN-PAGE was performed using the NativePAGE™ Novex® Bis-Tris Gel System (Thermo fisher Scientific, USA) according to the manufacturer’s protocol. CC-GFP and the empty vector were transiently expressed in *N. benthamiana* leaves individually using *Agrobacteria*. Plant samples were collected 36 h post inoculation and proteins were extracted for subsequent native PAGE analysis.

### Sequence analysis and structural homology modelling

The amino acid sequences of CC domains from Tsw and ZAR1 were aligned using ClustalW, and then edited by the ESPript (https://espript.ibcp.fr/ESPript/cgi-bin/ESPript.cgi) web server. Homology structure modelling was performed using PHYRE (Protein Homology/analogY Recognition Engine web server; http://www.sbg.bio.ic.ac.uk/~phyre2/html/page.cgi?id=index), with the template of ZAR1 (PDB: 6j5t). The structural models were displayed using PyMOL 2.4.1.

### RT-quantitative PCR

Total RNA from host and virus was extracted from *N. benthamiana* or pepper plant leaves using the Plant Mini Kit (Qiagen, 74904, Germany), and 2 µg of total RNA from each sample was reverse transcribed into cDNA using the TransScript One-Step gDNA Removal and cDNA Synthesis SuperMix (TransGen Biotech, AT311-03, China), according to the manufacturer’s instructions. RT–qPCR for both host mRNA and virus titre was performed on the CFX 96 system (Bio-Rad) using Thunderbird SYBR qPCR mix (TOYOBO, QPS-201), as described previously (Wu *et al.*, 2019). The primers used for mRNA detection of target genes by real-time PCR are listed in Supplementary Table S1. Data were normalized to *EF1α* expression by the cycle threshold (CT) 2–^ΔΔCT^ method. All experiments were repeated three times. Values are means ±SD; asterisks in RT–qPCR data panels indicate significant differences (Student’s *t*-test, *P<0.05; **P<0.01).

### Virus-induced gene silencing (VIGS)

For tobacco rattle virus (TRV)-based gene silencing, fragments of *NbSGT1* (Suppressor of G-two allele of Skp1), *NbRAR1* (Required for Mla12 resistance), and *NbHSP90* (Heat shock protein 90) were cloned from cDNA of *N. benthamiana* leaves and constructed into a pTRV2 vector (Qu *et al*., 2012). Subsequently, pTRV::SGT1, pTRV::RAR1, and pTRV::HSP90 plasmids were transformed into *A. tumefaciens* EHA105 strain. The indicated *A. tumefaciens* strains were co-infiltrated with *Agrobacterium* carrying a pTRV1 plasmid into 3-week-old *N. benthamiana* seedlings. Co-inoculation of pTRV1 and pTRV2 combination was used as a negative control. pTRV::PDS, the VIGS vector for silencing of *N. benthamiana PHYTOENE DESATURASE* (*PDS*) gene was used as positive control (Ye *et al*., 2009; Qu *et al*., 2012). Two weeks post-treatment, the gene knockdown efficiency of silenced plants was measured by RT–qPCR. At least six silenced plants per treatment were selected to inoculate with *Agrobacterium* carrying *35S:CC-GFP* plasmid. Later, cell death symptoms were observed and photographed under UV/white light. The experiment was repeated at least twice with similar results.

### Ion leakage assay

The ion leakage of treated leaf samples was measured according to a previous report with some modifications (Zhu *et al.*, 2017). Briefly, five leaf discs with a diameter of 5 mm were collected and floated on 5 ml of double-distilled water at 25 °C for 3 h, then the sample conductivity was measured and recorded as value A. Isolated leaf discs were treated at 95 °C for 25 min, and the value B of the corresponding samples were further measured when the solution cooled to 25 °C. The final conductivity was expressed as a percentage of the ion leakage=(value A/value B)×100.

### Disease index

Plant disease index is a standard method to quantify the disease incidence and severity for a population of plants (Cardoso *et al.*, 2010; H. Wang *et al*., 2015). We categorized the severities of TSWV-caused disease on *N. benthamiana* plants into six grades: 0, 1, 2, 3, 4, and 5. The grade 0 point indicates no disease symptom on the plant and grade 5 indicates dying plants after infection. Grade 1 to 4 is rated by disease symptom severity (Supplementary Fig. S1). The corresponding disease index was calculated as follows: Disease index=∑ (number of plants in each grade × each grade score)/ (total number of plants investigated × the maximum grade score) × 100.

### Data analysis

Differences in gene expression were determined by Student’s *t*-tests. A significant level of 0.05 was used for statistical analysis. One-way analysis of variance (ANOVA) followed by Duncan’s multiple range test were also used for data analysis. All statistical tests were carried out with GraphPad Prism. The relative fluorescence intensity of the cell death was estimated by ImageJ. In detail, the fluorescence channel of cell death images was split to measure the means of grey value, which indicated the fluorescence intensity (FI). Then the value was normalized to relative protein expression level (PE) observed in western blots. PE was estimated by ImageJ. The final calculating formula is as follows: The relative fluorescence intensity=[FI (sample) – FI (background)]/ [PE (sample)/ PE (loading)].

## Results

### The coiled-coil domain of Tsw triggers cell death

**Fig. 1.**
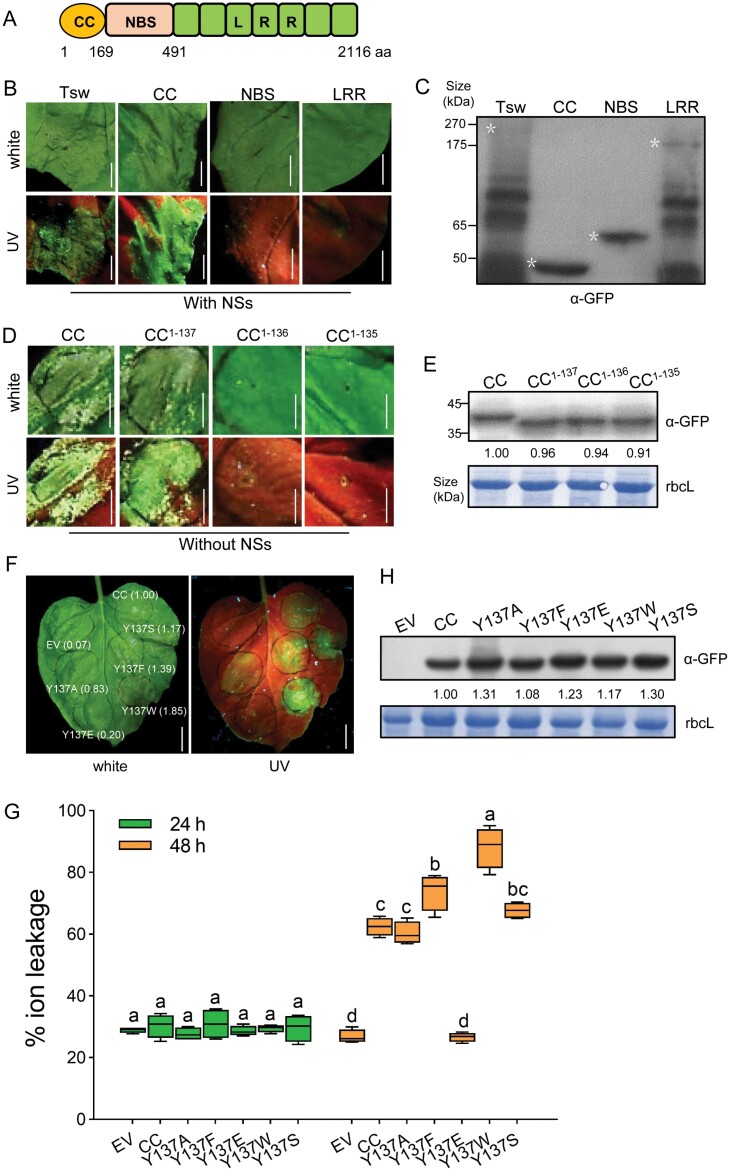
The coiled-coil domain of Tsw is sufficient for cell death induction *in planta*. (A) Schematic diagram of Tsw. CC, coiled-coil domain; LRR, leucine-rich repeat; NBS, nucleotide-binding site. (B) Cell death phenotypes of codon-optimized synthetic *Tsw* (*Tsw-GFP*) or domain truncations (*CC-GFP*, *NBS-GFP*, and *LRR-GFP*) co-infiltrated with *NS*s in *N. benthamiana* leaves. Photographs were taken 3 days (for domain truncations) or 7 days (for full-length Tsw) post-inoculation (dpi). Images under white and UV light are shown. Scale bars=0.5 cm. (C) Proteins were detected by immunoblotting with monoclonal anti-GFP antibody (α-GFP). GFP beads were used to enrich Tsw-GFP and LRR-GFP proteins. The asterisks indicate the expected protein. (D) Cell death phenotypes of CC domain truncations (CC-GFP, CC^1-136^-GFP, and CC^1-137^-GFP) in *N. benthamiana* leaves. Photographs were taken 3 dpi. Images under white and UV light are shown. Scale bars=0.5 cm. (E) Proteins were detected by immunoblotting with monoclonal α-GFP antibody. Coomassie brilliant blue (CBB) stained bands of the large subunit of rubisco (rbcL) were used as a loading control. The CC domain and its mutants bands were quantified by ImageJ software, the wide type CC was normalized to 1.00. (F) Cell death phenotypes (at 2 dpi) of CC^Y137^ point mutants (as GFP fusions) transiently infiltrated in *N. benthamiana* leaves. The numbers in parentheses represent the relative fluorescence intensity normalized to the relative protein expression level. The relative fluorescence intensity of cell death was estimated by ImageJ. Scale bars=1 cm. (G) Quantification of CC^Y137^ point mutant-induced cell death in *N. benthamiana* leaves, via ion leakage assay. Electrolyte leakage of leaf disks were measured at 24 h and 48 h after agroinfiltration (*n*=4; lower case letters indicate significant differences between samples; *P*<0.05, one-way ANOVA followed by Duncan’s multiple range test). (H) Accumulation of proteins detected by immunoblotting with α-GFP antibody. The CC and CC^Y137^ point mutant bands were quantified by ImageJ software; the wide type CC was normalized to 1.00 (relative values are indicated). Stained bands of rbcL were used as a loading control. In (B), (D), and (F), the agroinfiltrated leaves were illuminated under normal light (white) or a long-wavelength UV lamp.

Testing the potential for cell death-mediated disease resistance is the easiest method widely applied in plant NLR functional studies. To fully understand the mechanism of pepper NLR protein Tsw-triggered plant tospovirus defence, we first examined if Tsw possesses a single signalling domain to induce cell death and pathogen resistance. Tsw is a 2116 aa NLR protein initially identified from *Capsicum chinense* accessions (Kim *et al.*, 2017). Through sequence alignment with well-characterized NLRs, three domains were identified from Tsw protein, a coiled-coil (CC) domain (1–169 aa), a central nucleotide-binding site (NBS) domain (170–491 aa) and a COOH-terminal leucine-rich repeat (LRR) domain (492–2116 aa; Fig. 1A). Accordingly, we tested the capacity of each domain to induce visible cell death in the presence and absence of the viral effector NSs when transiently expressed in the heterologous host *Nicotiana benthamiana*. As seen in Fig. 1B, the CC domain (1–169 aa) was sufficient to trigger cell death in *N. benthamiana* leaves to degrees comparable to those seen with full-length Tsw in the presence of the viral effector NSs. A western blot analysis confirmed the correct expression of GFP fused with each domain of Tsw (Fig. 1C). Interestingly, the CC domain also has the activity to trigger cell death alone in the absence of NSs (Supplementary Fig. S2). The co-expression of either NBS or LRR domains of Tsw strongly abolished the death-inducing activity of the Tsw CC domain (Supplementary Fig. S3A), which could be explained by a direct inter-domain interaction (Supplementary Fig. S3B). These results indicated that the CC domain is the main signalling executor of Tsw to induce cell death.

The length of the CC domain and N/C-terminal tags may affect CC-triggered cell death. A series of deletion analysis further defined the minimal cell death-inducing fragment of Tsw as 1–137 aa (CC^1–137^). This fragment was even more potent to induce cell death than that of full-length Tsw. Importantly, the cell death induction ability by CC^1–137^ was fully abolished when Y^137^ was deleted within the hydrophobic rich motif F135-C136-Y137 (Fig. 1D). There was no obvious difference in protein accumulation among various mutants (Fig. 1E). We noticed that certain residues located in the hydrophobic groove of other NLRs also play key roles in their cell death activity, like Ile136 in ZAR1 (Cesari *et al.*, 2016; Wang *et al.*, 2019a). Based on the different performance of CC^1–137^ (autoactive) and CC^1–136^ (inactive) in cell death assays (Fig. 1D), we introduced several point mutations at Tyr137 within the CC domain (1–169 aa fragment) to test their efficacy in inducing cell death. Intriguingly, substitution of Tyr137 with a non-polar tryptophan residue (Y137W) strongly enhanced the capacity of the CC domain to induce cell death without changing CC protein accumulation (Fig. 1F–H), as indicated by the enhanced electrical conductivity of ions in the solution, reflecting that the degree of membrane damage was intensified (Fig. 1G). These results support a critical role of the hydrophobic groove, especially the Tyr^137^ residue, in inducing cell death for the Tsw protein.

### Coiled-coiled domain localized on the plasma membrane

**Fig. 2.**
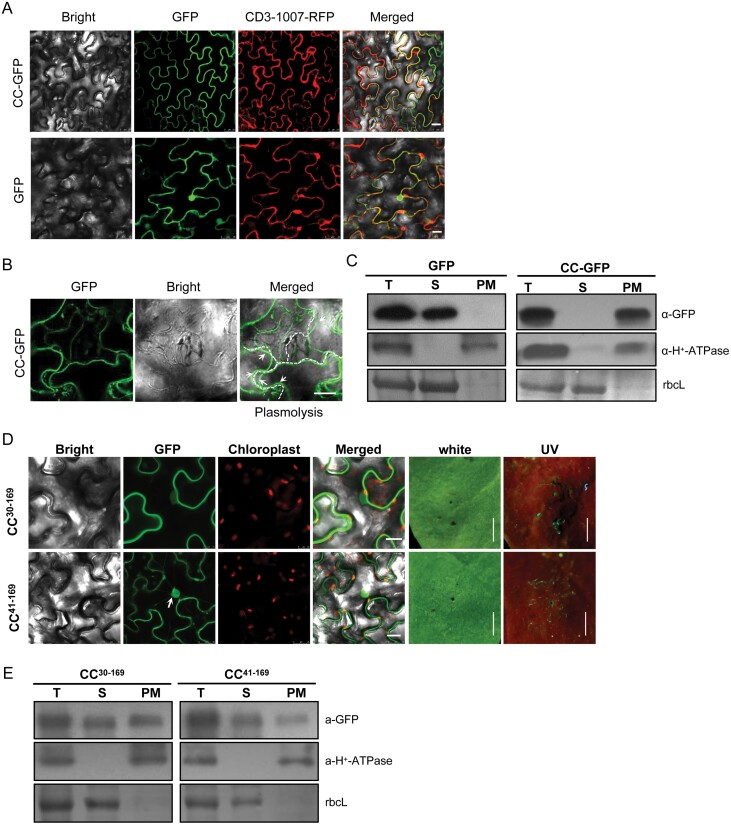
Tsw CC is localized to the plasma membrane. (A) Sub-cellular localization of CC-GFP and GFP empty vector control were observed in *N. benthamiana* at 2 days post-inoculation (dpi). Scale bars=25 μm. (B) GFP fluorescence was observed after cell plasmolysis in *N. benthamiana* leaves. Leaves were treated with 5% NaCl for 5–10 min to trigger cell plasmolysis. The cell wall is marked by dotted lines. Typical retracted plasma membrane is indicated by arrowheads. Scale bars=25 μm. (C) Total protein (T) was separated into soluble (S) and plasma membrane (PM) fractions. The H^+^-ATPase was used as a plasma membrane marker. Antibodies against GFP and H^+^-ATPase were used. (D) Sub-cellular localization of CC N-terminal deletion mutants (CC^30-169^-GFP, CC^41-169^-GFP) were observed by confocal microscopy, and cell death phenotype was photographed under white and UV light. Scale bars for confocal images=25 μm. Scale bars for leaf images=0.5 cm. (E) Immunoblotting of CC mutants separated into soluble (S) and plasma membrane (PM) fractions. Antibodies against GFP and H^+^-ATPase were used. In (A), (B) and (D), the GFP, RFP, and chloroplast auto-fluorescence was observed under 488 nm, 532 nm, and 561 nm, respectively.

The sub-cellular localization of NLR proteins are pivotal for their proper functioning and full activity (Qi *et al.*, 2012; Kawano *et al.*, 2014; Cesari *et al.*, 2016; Wang *et al.*, 2019a). We next set out to characterize the sub-cellular localization of Tsw and its derivatives. We expressed the CC-GFP fusion protein in leaf cells of *N. benthamiana*. Confocal microscopy results showed that the fluorescence signal of CC-GFP fusion protein was fully co-localized with the plasma membrane marker CD3-1007-RFP (Nelson *et al.*, 2007), while GFP alone had a partial fluorescence signal in the nucleus (Fig. 2A). Meanwhile, plasmolysis analysis further suggested the plasma membrane localization of CC-GFP (Fig. 2B). The fluorescence signals in the cytoplasm and plasma membrane of *N. benthamiana* cells were indistinguishable. To confirm this membrane association of Tsw CC, we performed a cellular compartment fraction assay on the GFP and CC-GFP expressing leaf cells of *N. benthamiana*. As expected, CC-GFP was exclusively detected in the plasma membrane fraction, and GFP control was mostly detected in soluble fractions, which further confirmed the plasma membrane localization of the CC domain (Fig. 2C).

N/C-terminal tags may affect CC-mediated cell death. We observed that the N-terminal amino acid sequence is essential for Tsw CC function, and the N-terminal GFP fusion, but not the C-terminal fusion affects cell death-induction ability of Tsw CC (Supplementary Fig. S4). We further verified whether the N-terminal region of the CC domain is involved in its activity by constructing several truncations of the CC domain, according to its predicted protein secondary structure. Through transient expression in *N. benthamiana* leaves, we found that its plasma membrane anchoring activity is greatly dampened, proving that the N-terminal region of CC is impaired. The sub-cellular locations of CC^30–169^-GFP and CC^41–169^-GFP were partially changed to the nucleus (Fig. 2D). The same results were observed in a membrane fractionation assay, and the above-described N-terminal deletion mutants of CC were partially soluble (Fig. 2E). Accordingly, these N-terminal truncated CC mutants abolished cell death induction in *N. benthamiana* (Fig. 2D). Interestingly, an N-terminal fragment (1–25 aa) of CC could partially re-localize GFP to the plasma membrane, as indicated by the membrane fractionation assay results of GFP and CC^1-25^-GFP proteins (Supplementary Fig. S5). These results suggested that the N-terminal region of the CC domain is essential for its association with the plasma membrane, and the ability to trigger cell death.

### A hydrophobic module of Tsw is associated with its cell death induction

**Fig. 3.**
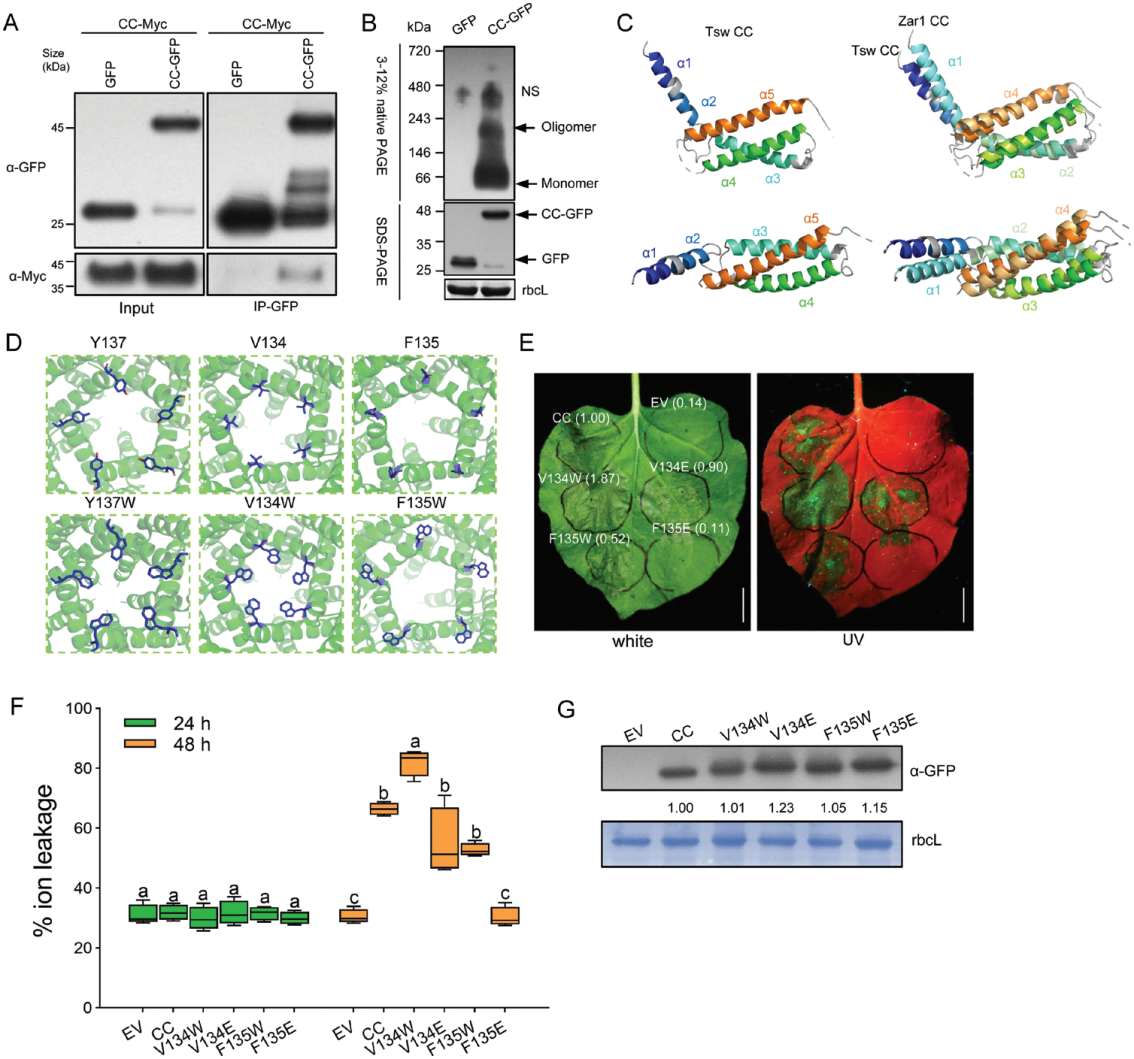
The hydrophobic module is essential for cell death induction of Tsw. (A) Differentially epitope tagged CC proteins (CC-Myc, CC-GFP) and empty vector (GFP) were transiently expressed in *N. benthamiana* in the indicated combinations (*35S:CC-GFP* + *35S:CC-Myc*, and *35S:GFP* + *35S:CC-Myc*). Three days post infiltration, samples were collected for co-immunoprecipitation analysis. Tagged proteins were enriched with GFP beads, and detected with anti-Myc (α-Myc) and anti-GFP (α-GFP) antibodies. (B) CC oligomerization state, as detected by BN-PAGE. Arrows point to oligomers and monomers. NS, nonspecific bands (upper image). The protein expression level and loading control were detected by SDS-PAGE and rbcL, respectively (bottom image). (C) Predicted structure model of Tsw CC domain. Structure modelling of Tsw CC domain based on the structure of ZAR1 (PDB ID: 6J5T). The left panels indicate the Tsw CC domain and labelled α-helix with different colours. The right panels indicate the Tsw CC predicted structure mapped to ZAR1 CC structure. The α-helix of ZAR1 CC was marked in different colours. (D) Predicted pentameric structural model of N termini of Tsw and Tsw mutants using the reference model for ZAR1 (PDB ID: 6J5T). The changed residues are indicated in blue. (E) Cell death phenotypes (at 2 dpi) of CC point mutants transiently infiltrated into *N. benthamiana* leaves. The agroinfiltrated leaves were illuminated under normal (white) light and a long-wavelength UV lamp. The fluorescence intensity of cell death was estimated by ImageJ. Scale bars=1 cm. (F) Ion leakage quantification of CC point mutants-induced cell death in *N. benthamiana* leaves at 24 h and 48 h after agro-infiltration (*n*=4; lower case letters indicate significant difference between samples; *P*<0.05, one-way ANOVA, followed by Duncan’s multiple range test). (G) Confirmation of protein expression by immunoblotting with α-GFP antibody. Staining of the large subunit of rbcL provided a loading control.

To decipher the underlying mechanism of this single signalling CC domain, we next analysed the oligomerization state of the CC domain *in vivo* using protein extracts from infiltrated *N. benthamiana* leaves. The CC domain could form oligomers, as evidenced by co-immunoprecipitation (co-IP) and BN-PAGE (Fig. 3A, B). We then analysed the structure of the Tsw CC domain by protein homology modelling using the PHYRE web server. Homology modelling results showed that the structure of the Tsw CC domain aligned well with that of the ZAR1 CC domain (Fig. 3C; Supplementary Fig. S6). The above results showed that hydrophobic amino acid Tyr137 is critical for its full function in cell death induction, and the CC^Y137W^ mutant has enhanced cell death activity (Fig. 1F). We observed that the hydrophobic module is near the central pore with a putative ion permeation path, and therefore we next queried the relationship between the structure formed by hydrophobic amino acids and Tsw CC-induced cell death. We analysed the modelled oligomeric structure of Tsw, and found that the residues around Trp137 in the Tsw CC^Y137W^ domain form a more compact hydrophobic groove than that in wild type Tsw (Fig. 3D). Considering the increased cell death caused by the CC^Y137W^ domain *in planta* (Fig. 1F), we assumed that the change from Tyr to Trp causes a superhydrophobic funnel-like structure that further stabilizes its oligomerization. To further verify this hypothesis, we analysed other residues within the hydrophobic region of the CC domain (132–137 aa). Notably, the oligomerized Tsw CC structure indicated that the mutation of Val^134^ to Trp^134^ confers the 134^th^ residue to have extensive contact with its neighbouring residues (Fig. 3D, second panel). In contrast, substitutions with other residues within the hydrophobic region (e.g. Phe135) lead this residue to face outside of the funnel-like structure (Fig. 3D, third panel). The follow-up transient expression assay and ion leakage data showed that CC^V134W^ indeed enhanced cell death, though a similar protein level accumulated with the WT CC control, consistent with the predicted Tsw oligomer structure (Fig. 3E–G). These results suggested that the strategy of structure-assisted improvements on the CC domain for NLR proteins against pathogens is feasible. Moreover, the amino acids in the hydrophobic groove affect the activity of the CC domain irrelevant of its localization, since the loss-of-function mutants of CC still localize on the plasma membrane (Supplementary Fig. S7).

### Tyrosine to tryptophan substitution in the hydrophobic groove of Tsw enhanced its resistance to TSWV

**Fig. 4.**
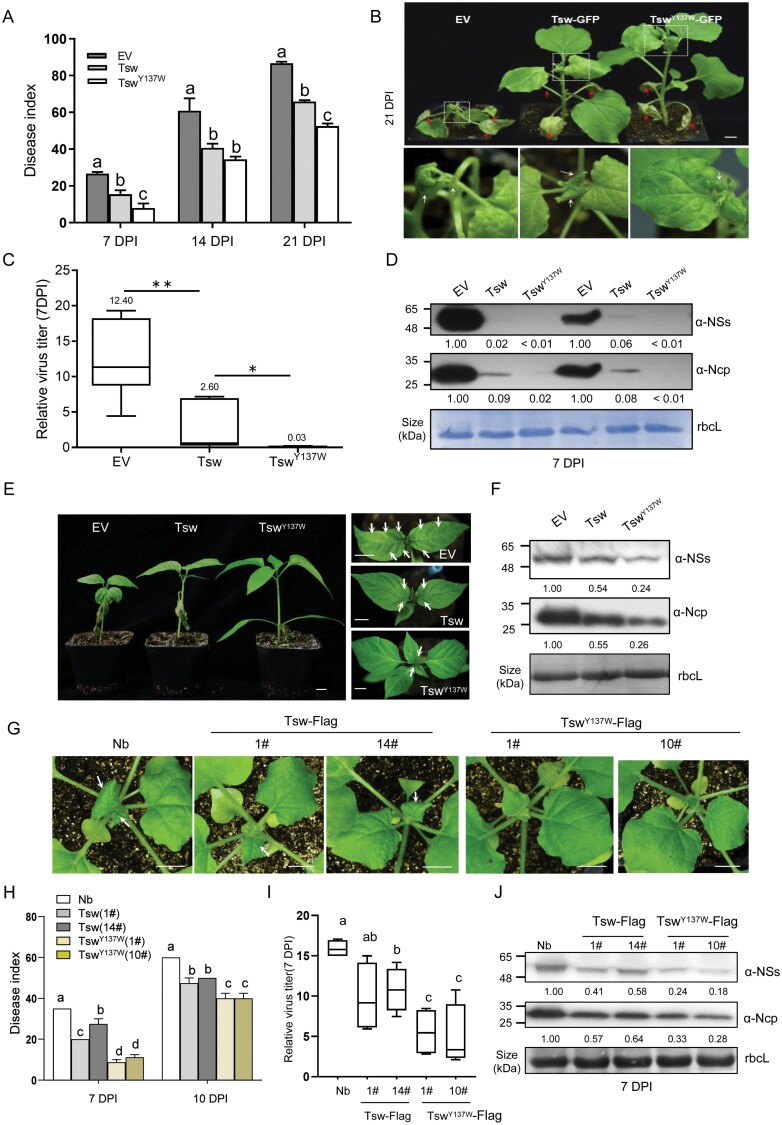
The gain-of-mutation Tsw^Y137W^ enhances plant resistance to TSWV. (A) Disease index of TSWV-infected *N. benthamiana* plants was calculated at 7, 14, and 21 days post-inoculation (dpi) (*n*=4; lower case letters indicate significant difference between samples; *P*<0.05, one-way ANOVA followed by Duncan’s multiple range test). (B) *Agrobacterium* harbouring the empty vector (EV), *Tsw-GFP*, or *Tsw*^*Y137W*^*-GFP* constructs were transiently infiltrated into *N. benthamiana* leaves, followed by inoculation with TSWV of infiltrated leaves 2 d later. Systemic symptoms of TSWV-inoculated *N. benthamiana* were photographed 3 weeks post-inoculation. White arrows point to typical symptoms; red arrows indicate inoculated leaves. Scale bars=1 cm. (C) Relative TSWV titre in *N. benthamiana* at 7 dpi, as determined by RT–qPCR of *Ncp* expression. Values are means ±SD; *n*=6, *P<0.05, **P<0.01, by Student’s *t*-test. (D) TSWV abundance in plants at 7 dpi, as detected with antibodies against NSs or Ncp. Viral NSs or Ncp bands were quantified by ImageJ software, the viral accumulation in EV transiently expressed plants was normalized to 1.00. (E) The empty vector (EV), *Tsw-Flag*, or *Tsw*^*Y137W*^*-Flag* were transiently infiltrated into pepper leaves, followed by inoculation with TSWV-YN on infiltrated leaves 2 d later. Systemic symptoms of TSWV-inoculated pepper were photographed 14 dpi. Scale bars=1 cm. (F) TSWV abundance in pepper at 14 dpi, viral NSs or Ncp were detected with antibodies against NSs or Ncp, and were quantified by ImageJ software and the values obtained are indicated; the viral accumulation in EV transiently expressed plants was normalized to 1.00. (G) *Tsw-*Flag and *Tsw*^*Y137W*^*-*Flag transgenic lines were inoculated with TSWV. Systemic symptoms of TSWV-inoculated *N. benthamiana* were photographed 7 dpi. White arrows point to typical symptoms. Scale bars=1 cm. (H) Disease index of TSWV-infected *N. benthamiana* plants was investigated within 10 dpi, and the individual disease index was calculated (*n*=4; lower case letters indicate significant difference between samples; *P*<0.05, ANOVA followed by Duncan’s multiple range test). (I) Relative TSWV titre in *N. benthamiana* at 7 dpi was determined by RT–qPCR of *Ncp* expression. Values are means ±SD, *n*=6; *P*<0.05, ANOVA followed by Duncan’s multiple range test. (J) TSWV abundance in transgenic plants at 7 dpi, antibodies against NSs and Ncp were used. Viral NSs or Ncp bands were quantified by ImageJ software and the values obtained are indicated; the viral accumulation in non-transgenic plants was normalized to 1.00.

To test whether the hydrophobic residue substitution was functional in the full-length Tsw with enhanced immunity against TSWV, we generated a construct harbouring full-length *Tsw*^*Y137W*^*-GFP* and transiently expressed this ­gain-of-function mutant or WT Tsw control in *N. benthamiana* leaves, followed by mechanical inoculation with TSWV-YN at 2 d post-infiltration. The disease incidence of plants infected with TSWV was investigated, and the disease index recorded, to describe the average disease severity on the TSWV-infected plants (Supplementary Fig. S1). Plants expressing *Tsw*^*Y137W*^*-GFP* exhibited greater resistance to TSWV infection than those expressing *Tsw-GFP*, as indicated by their much weaker disease symptoms and the dramatically reduced disease index (Fig. 4A, B). Consistent with results of cell death induction, the expression of *Tsw*^*Y137W*^*-GFP* confers plants with much stronger disease resistance, as indicated by much lower levels of viral RNA and proteins in newly emerged leaves than those expressing *Tsw-GFP* (Fig. 4C, D). To confirm this enhanced virus resistance observed in the model plant *N. benthamiana*, we transiently expressed WT *Tsw* and the gain-of-function mutant Tsw^Y137W^ in a pepper cultivar sensitive to TSWV infection. Consistently enhanced resistance to TSWV infection was also observed on pepper plants expressing *Tsw*^*Y137W*^ than those expressing *Tsw* (Fig. 4E, F). These data confirmed that Tsw^Y137W^ confers improved resistance to TSWV infection in different plant species.

Moreover, to further test the enhanced resistance of Tsw^Y137W^ to TSWV, we generated several transgenic *N. benthamiana* plants stably transformed with *Tsw-Flag* and *Tsw*^*Y137W*^*-Flag* constructs. Dozens of T_0_ transgenic *N. benthamiana* plants were generated either expressing Tsw-Flag or Tsw^Y137W^-Flag proteins. Molecular characterization including gene expression and protein accumulation of Tsw-Flag and Tsw^Y137W^-Flag was performed to identify transgenic plants with similar expression level of Tsw-Flag (lines #1 and #14) and Tsw^Y137W^-Flag (lines #1 and #10; Supplementary Fig. S8). Each transgenic line of Tsw-Flag and Tsw^Y137W^-Flag was further tested to ascertain that T_1_ progeny transgenic plants were resistant to the TSWV-YN strain. Milder symptoms were observed in T_1_ plants ectopically expressing *35S:Tsw*-*Flag*, and no obvious disease symptoms were seen on *35S:Tsw*^*Y137W*^*-Flag* plants Fig. 4G; in contrast, typical leaf curling caused by TSWV infection was observed in systemic leaves of mock plants at 7 dpi. Notably, *35S:Tsw*^*Y137W*^-*Flag* transformants exhibited enhanced resistance compared with *35S:Tsw*-*Flag* transformants, even though Tsw showed comparable expression levels (Fig. 4H; Supplementary Fig. S8). The following RT–qPCR and western blot analysis confirmed the lower virus titre and accumulation of viral Ncp and NSs proteins in infected *35S:Tsw*^*Y137W*^ transformants, than in *35S:Tsw* transformants (Fig. 4I, J). These results demonstrated that the hydrophobic groove of Tsw can be engineered to improve the pepper NLR Tsw when expressed in a heterologous plant like *N. benthamiana*.

### The hydrophobic groove conserved in an autonomous NLR clade in seed plants

**Fig. 5.**
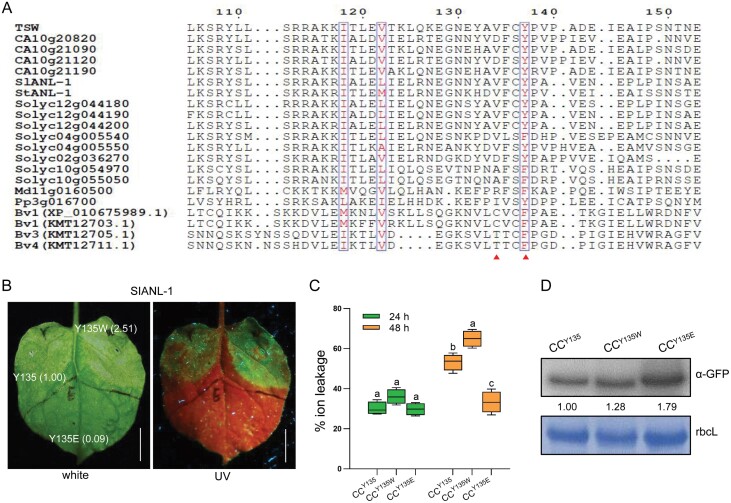
A conserved Tyr^135^ residue is essential for cell death induction of tomato SlANL-1 domain. (A) Alignment of several CC domains from NLRs in the ANL (ancient and autonomous NLR) clade. The red triangle indicates the conserved amino acid matching Val134 and Tyr137 in Tsw. Residues considered as highly similar are framed in boxes. (B) Cell death phenotypes induced by transient infiltration of the SIANL-1 CC domain (CC^Y135^-GFP) and its two variants (CC^Y135W^-GFP and CC^Y135E^-GFP) in *N. benthamiana* leaves. The agroinfiltrated leaves were illuminated under normal (white) light and a long-wavelength UV lamp at 2 days post-inoculation. The fluorescence intensity of cell death was estimated by ImageJ. Scale bars=1 cm. (C) Ion leakage assay of leaf disks from SIANL-1 CC mutants expressed in *N. benthamiana* was measured at 24 h and 48 h after agroinfiltration (*n*=4; lower case letters indicate significant difference between samples; *P*<0.05, one-way ANOVA followed by Duncan’s multiple range test). (D) Protein accumulation was measured by immunoblotting with GFP antibody. The CC mutant bands were quantified by ImageJ software and the obtained values are shown; the intensity of CC^Y135^ band was normalized to 1.00. Coomassie blue stained the large subunit of rubisco (rbcL) that was used as a loading control.

Tsw was recently classified as a member of the well-conserved autonomous NLR (ANL) clade, which is present in all tested seed plants (Lee *et al.*, 2021). To determine how common the strategy of hydrophobic groove-mediated immunity enhancement might be, we aligned the protein sequences of CC domains from members of the ANL clade from taxonomically distant plants, including *Solanaceae*, *Rosaceae*, and *Chenopodiaceae*. Indeed, the hydrophobic groove showed a highly conserved pattern, with a conserved aromatic amino acid (Tyr or Phe) at the position corresponding to Tsw Tyr137, while the Val134 exhibited a slightly changeable pattern (Fig. 5A). We randomly selected the *Solanum lycopersicum* ANL member SlANL-1 to verify our above result. Accordingly, we generated one loss-of-function substitution (CC^Y135E^-GFP) and one gain-of-function substitution (CC^Y135W^-GFP), and transiently infiltrated the resulting constructs in *N. benthamiana* leaves for functional verification. The loss-of-function substitution (CC^Y135E^-GFP) failed to induce cell death, while the gain-of-function substitution (CC^Y135W^-GFP) exhibited a more severe cell death phenotype than the control CC^Y135^-GFP, accompanied by higher ion conductivity (Fig. 5B, C). A western blot analysis confirmed the correct expression of GFP-fused CC proteins of SlANL-1 (Fig. 5D). These results indicated that NLRs from ANL members could also benefit from hydrophobic groove improvement, regardless of their natural host.

### Host SGT1-dependent cell death induction for Tsw coiled-coil domain

**Fig. 6.**
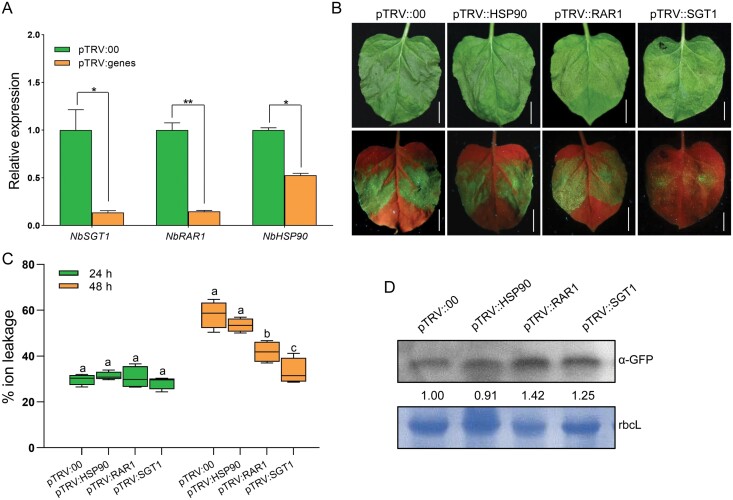
The autoactivity of Tsw CC *in planta* requires plant immunity signalling component SGT1. (A) The relative expression of the silenced genes *NbSGT1*, *NbRAR1* and *NbHSP90* in *N. benthamiana*. The values were normalized to expression level of *NbEF1α*. Values are means ±SD (*n*=4; *P<0.05, **P<0.01, Student’s *t*-test). (B) *A. tumefaciens* carrying *35S: CC-GFP* plasmids were infiltrated into *SGT1*, *RAR1*, and *HSP90* -silenced *N. benthamiana* leaves. Cell death phenotypes under white/UV light are shown. Scale bars=1 cm. (C) Quantification of cell death induction of CC-GFP in *SGT1*-, *RAR1*-, and *HSP90*-silenced *N. benthamiana*. Electrolyte leakage of leaf disks from *N. benthamiana* were measured at 24 h and 48 h after agroinfiltration (*n*=4; lower case letters indicate significant difference between samples; *P*<0.05, one-way ANOVA followed by Duncan’s multiple range test). (D) Protein accumulation of CC-GFP determined by western blot. Total soluble protein was detected by monoclonal antibody against GFP. The CC bands were quantified by ImageJ software and the intensity of the bands are indicated; the CC accumulation in pTRV::00 infected plants was normalized to 1.00. Coomassie-stained rbcL was used as a loading control.

In plants, molecular chaperone components SGT1, RAR1, and HSP90 are essential for plant resistance mediated by a number of NLR proteins (Botër *et al.*, 2007). To gain insight into the cell death signalling mechanism of Tsw, we tested whether SGT1, RAR1, and HSP90 participated in Tsw CC-induced virus resistance. We conducted cell death assays on these plant cells with gene knock down expression by TRV-based VIGS. The RT–qPCR results demonstrated that *NbSGT1*, *NbRAR1*, as well as *NbHSP90* transcripts were dramatically decreased, compared with those of the vector control plants (Fig. 6A). The Tsw CC-induced cell death was dramatically compromised in *SGT1*-silenced plants (Fig. 6B). This conclusion was further supported by results from the ion leakage assay at 48 h (Fig. 6C). Moreover, we also measured the accumulation of Tsw CC domain in *HSP90*, *SGT1*, and *RAR1*-silenced plants (Bhattarai *et al.*, 2007; Zhang *et al.*, 2010); western blot analysis confirmed the correct expression of GFP-fused CC proteins (Fig. 6D). Sub-cellular compartment distribution of CC-GFP protein showed no obvious differences between WT and these genetically defective plants (Supplementary Fig. S9), suggesting that *HSP90*, *SGT1*, and *RAR1* are not necessary for the steady state levels and sub-cellular localization of the Tsw CC domain.

## Discussion

Once activated, NLRs mediate disease resistance and induce cell death simultaneously. However, the precise connection between disease resistance and cell death is largely unknown. Here, we discovered that the hydrophobic groove in CC ­domain can be explored to enhance plant NLR-mediated resistance. These results provide new targets to optimize plant immunity mediated by Tsw and other ANL proteins.

### Coiled-coil domain individually induces cell death *in planta*

Each plant genome encodes hundreds of NLR proteins. Most NLRs possess an N-terminal signalling domain that induces visible cell death in heterologous plants, including TIR (Toll-interleukin-1 receptor) domains from RBA1 (Response to the bacterial type III effector protein HopBA1), RPS4 (Resistance *to Pseudomonas syringae* 4), L6 (flax polymorphic L locus protein 6), and CC domains from ZAR1, MLA10 (polymorphic barley mildew A 10) , Sr33 (Stem rust resistance gene 33), Sr50 (Stem rust resistance gene 50), NRG1 (N requirement gene 1), RPM1 (Resistance to *Pseudomonas maculicola* 1), and RP1 (Resistance to *P. sorgbi* 1; Qi *et al.*, 2012; G.F. Wang *et al*., 2015; Casey *et al.*, 2016; Nishimura *et al.*, 2017; Zhang *et al.*, 2017; Horsefield *et al.*, 2019; Baudin *et al.*, 2020; Bai *et al*., 2012). Recently, the N-terminal amphipathic helix in the CC domain of ZAR1 was shown to form a funnel-shaped structure that is required for plasma membrane-associated cell death (Wang *et al.*, 2019a, b). A few functionally important motifs of the CC domain have been identified from CNLs, such as MADA and α1 helix (Adachi *et al.*, 2019; Lee *et al.*, 2021). Tsw is a singleton and autonomous NLR, carrying an autoactive CC domain in pepper (Fig. 1B), with a minimal unit to induce cell death *in planta* (Fig. 1D). Our data strongly support the concept that Tsw CC domain has a plasma membrane-anchoring ability, consistent with the predicted structural model of Tsw (Fig. 2A-C). The importance of plasma membrane localization has been addressed in several CNLs (Zhou *et al.*, 2015; Chen *et al.*, 2017; El Kasmi *et al.*, 2017; Wang *et al.*, 2020). Data presented here also strongly suggested that the plasma membrane of Tsw has significant roles in immunity against TSWV (Fig. 2D, E), such as the Ca^2+^ influx induction of the CC domain, as evidenced by the Tsw CC domain-mediated cell death being dependent on Ca^2+^ (Supplementary Fig. S10).

### Hydrophobic module regulates coiled-coil-mediated cell death

The hydrophobic region of the CC domain affects the formation of ZAR1 resistosome, and thereby NLR-induced immunity (Wang *et al.*, 2019a). The Phe^135^, Ile^136^, and Thr^137^ residues are centred at the ZAR1 oligomerization interface, which make extensive hydrophobic contacts with their neighbouring residues, and the ZAR1^I136E^ mutant compromised its cell death induction activity. Consistently, the corresponding Val^134^, Phe^135^, Cys^136^, and Tyr^137^ residues of Tsw CC play an important role in cell death, and a mutant of Tyr^137^ can potentially increase the Tsw-mediated virus resistance to TSWV in *N. benthamiana* (Fig. 4). We confirmed the self-association ability of Tsw CC (Fig. 3A, B). Unlike the Ile^136^ of ZAR1, there are no amino acid residues near Tyr^137^ that provide hydrogen bonds. Interestingly, Tyr^137^ in every monomer of Tsw is close to each other and could form strong hydrophobic interactions to stabilize the funnel-shaped structure. The substitution of Tyr^137^ to Glu^137^ lost a hydrophobic environment and resulted in a loss-of-function in triggering cell death (Fig. 1F). As visible from the predicted structure of Tsw, hydrophobic interaction is enhanced by the substitution of Tyr^137^ to Trp^137^ mutant, leading to a more stable funnel-shaped structure (Fig. 3D). According to this principle, we substituted Val^134^ (hydrophobic force enhanced) and Phe^135^ (hydrophobic force weakened) with Trp. The cell death phenotypes further proved the concept that the hydrophobic groove of NLRs is a good target for improvement, and the structure-assisted method could be very useful (Fig. 3D, E).

The hydrophobic region (122–134 aa) of the Sr33 cluster is important for self-association and auto-activity of the CC domain (Cesari *et al*., 2016). There are some similarities among these different NLRs. The region (134–137 aa) of Tsw CC domain is also enriched in hydrophobic residues and important for its auto-activity (Supplementary Fig. S7). Tsw was classified in the ANLs cluster, and the R protein in this novel cluster shared several features, such as carrying an autoactive CC domain and localizing to the plasma membrane (Lee *et al.*, 2021). Consistently, the gain-of-function mutant for R protein improvement was further validated in the tomato Tsw paralogous R protein (Fig. 5B); *Tsw*-mediated resistance to TSWV was specific, as Tsw transgenic plants showed no obvious effect when infected by turnip mosaic virus (TuMV), another RNA virus Supplementary Fig. S11). It would be valuable to investigate whether the key function of amino acids in the ­hydrophobic groove is also a common feature of NLRs in this cluster.

As one of the two resistance genes against TSWV, *Tsw* is not very potent because of its narrow disease resistance spectrum, and it could be overcome with temperature and rapidly evolving pathogens (Turina *et al.*, 2016). We observed that transgenic expression of *Tsw* in *N. benthamiana* and transient expression in pepper (*C. annuum*) could only induce moderate resistance. Several reasons may explain this observation. One reason could be the low expression level in both test systems. Several reports have shown that *R* gene overexpression results in enhanced disease resistance, and positively correlates with their expression levels (Oldroyd and Staskawicz, 1998; Stokes *et al.*, 2002; Tang *et al.*, 1999). Moreover, some newly emerging TSWV isolates were able to break the *Tsw* resistance gene-mediated plant defence, including a tomato isolate (TSWV-YN18) and a tobacco isolate (TSWV-YN53; Jiang *et al.*, 2017). TSWV NSs have been found to be the key determinant for breaking resistance in Tsw-carrying pepper plants (Margaria *et al.*, 2007). The amino acid sequence of NSs in our study (TSWV-YN) has a very high sequence identity with that of TSWV-YN18 and TSWV-YN53 (99.14% and 99.36%, respectively). The TSWV-YN isolate used in our study might be a resistance-breaking (RB) strain. Nevertheless, at a similar low expression level, the Tsw^Y137W^ mutant enhanced resistance to TSWV in tobacco and pepper plants under the same Tsw construct background, which suggests that it is a feasible way of structure-assisted improvements of NLR against pathogens.

Artificial evolution of *R* genes in crops will provide more valuable resources for breeding. Whether the module shown in the current study is workable in fields needs further research on TSWV-sensitive gene editing of crops. Thus, it is necessary to carry out base-specific gene editing to improve resistance in pepper varieties that contain the *Tsw* gene. To develop transgenic plants in pepper is a challenging task, compared with that in other *Solanaceae* species, because of the lack of an effective transformation system for pepper and its low regeneration rate under *in vitro* conditions (Kothari *et al.*, 2010; Turina *et al.*, 2016). This limitation needs to be overcome in future research.

### Molecular regulation of Tsw-mediated cell death

Precise artificial *R* gene evolution requires a detailed understanding of activation of NLRs. Plant NLRs recognize pathogens both indirectly and directly to trigger immunogenic signals (Zhou and Zhang, 2020). This is the first molecular analysis of Tsw in TSWV immunity. Tsw is classified as a singleton NLR which is supposed to function as a single unit to confer full resistance (Lee *et al.*, 2021). Interestingly, we found that both the NBS and LRR (NBS-GFP and LRR-GFP) domains in Tsw could attenuate autoactivation of the CC domain. We confirmed the interaction between the NBS and CC domains in a co-IP assay (Supplementary Fig. S3B). These results indicated that Tsw could self-regulate, and its activation may be due to CC domain exposure, with the NBS domain inhibiting its activation.

In addition, epigenetic regulation, temperature, and phytohormones are also reported to associate with NLR-mediated plant immunity (Chung *et al.*, 2018; Li *et al.*, 2020; Yang *et al.*, 2020). Amongst these, as one of the important environmental factors, temperature has a modulating effect on many biological processes, especially to antiviral immunity (Qu *et al.*, 2005; Wang *et al.*, 2009). One of the drawbacks of some *R* genes is temperature sensitivity; they only provide resistance at a certain temperature range (Wang *et al.*, 2009; Lim *et al.*, 2014). *Tsw*-mediated resistance is reduced at 28 °C, and does not provide resistance at a temperature higher than 32 °C (de Ronde *et al.*, 2019). Mutations in the *R* gene sequence against tobacco mosaic virus provide improved resistance at high temperature (Whitham *et al.*, 1996), although the underlying mechanism is still elusive. Whether the enhanced TSWV resistance conferred by Tsw mutants (Tsw^Y135W^ and Tsw^Y137W^) could perform better under higher environmental temperature conditions still needs to be tested in the future.

### Effect of SGT1 on coiled-coil/Tsw function

The precise function of SGT1 is still a mystery as it is involved in several unrelated processes. Our data presented here provide some clues that Tsw conducts the resistance response via SGT1 (Fig. 6). Molecular chaperone components SGT1, RAR1, as well as HSP90 form a complex to contribute to lots of *R* gene-mediated resistance (Bhattarai *et al.*, 2007; Botër *et al.*, 2007; Shirasu, 2009; Zhang *et al.*, 2010). Several studies indicated that the steady state level of R protein depends on the chaperone activity of SGT1 and HSP90 (Takahashi *et al.*, 2003; Azevedo *et al.*, 2006); the chaperone function of SGT1 is required for *R* gene function in several plant-microbe interactions (Wang *et al.*, 2010). For example, SGT1 is reported to play a positive role in *R* gene-induced cell death. It is required by *Sw-5b*-mediated TSWV resistance, and *Mi-1*-mediated pathogen and pest resistance (Bhattarai *et al.*, 2007; Kadota *et al.*, 2010; Chen *et al.*, 2021). The interaction between SGT1 and other chaperones also contribute to the accumulation of NLR proteins and defence signalling (Kadota *et al.*, 2008). In this study, our results showed that CC protein accumulation and sub-cellular localization were unaffected in SGT1-silenced plants (Fig. 6D; Supplementary Fig. S9). Therefore, we hypothesize that SGT1 may not directly influence the function of Tsw-CC. Instead, SGT1 may act downstream of CC-mediated defence signal transduction. More research is required to underpin the underlying mechanisms.

Since it serves as a critical immune regulator, it is not surprising that SGT1 is often targeted by pathogens of plants and animals (Bhavsar *et al.*, 2013; Yu *et al.*, 2020). Despite that SGT1 plays a positive role in host and non-host resistance in *N. benthamiana*, the infection of some RNA viruses such as TSWV and potato virus X has been shown to be impaired in *SGT1*-silenced *N. benthamiana*, which is explained by the blocked function of viral movement protein (Ye *et al.*, 2012; Qian *et al.*, 2018).

In conclusion, we have developed more robust R proteins in a structure-assisted manner by optimizing the hydrophobic groove of ANL-type NLRs. Function- and structure-based reprogramming of *R* genes using biotechnological applications, such as genome editing provide valuable resources for breeding disease-resistant crops, especially those lacking robust and defined genetic resistance. Further genome editing on *Tsw-*harbouring pepper species, and overexpressing the Tsw^Y137W^ mutant in the genomes of non-pepper crops, will expand the feasibility and usefulness of this strategy.

## Supplementary data

The following supplementary data are available at *JXB* online.

Fig. S1. Symptoms scale of TSWV-infected *N. benthamiana*.

Fig. S2. Cell death phenotypes of codon-optimized synthetic *Tsw* (*Tsw-GFP*) or domain truncations (*CC-GFP*, *NBS-GFP*, and *LRR-GFP*) expressed in *N. benthamiana* leaves.

Fig. S3. Both NBS and LRR domains of Tsw attenuate the autoactivation of the CC domain.

Fig. S4. N-terminal free of fusion is critical for cell death induction of Tsw.

Fig. S5. CC^1-25^ fragment partially re-localizes cytoplasmic GFP protein onto the plasma membrane.

Fig. S6. Structure-based sequence alignment of Tsw CC and ZAR1 CC.

Fig. S7. The hydrophobic groove in Tsw CC domain is critical for its autoactivation.

Fig. S8. Detection of Tsw and TSW^Y137W^ transgenic *N. benthamiana* plants.

Fig. S9. Tsw CC domain is localized to the plasma membrane in *SGT1*-, *RAR1*-, and *HSP90*-silenced *N. benthamiana* plants.

Fig. S10. Cell death induced by Tsw CC is Ca^2+^ -dependent.

Fig. S11. Effect of *Tsw* transgenic plants on TuMV-GFP infection.

Table S1. DNA primers used in this study.

erac477_suppl_Supplementary_Figures_S1-S11_Table_S1

## Data Availability

All data supporting the findings of this study are available within the paper and within its supplementary data published online.
