# Supplementary material for: Structure–function analyses of coiled-coil immune receptors define a hydrophobic module for improving plant virus resistance
Source: J Exp Bot. 2022 Dec 6;74(5):1372–88. doi: 10.1093/jxb/erac477 (PMC10010612; doi:10.1093/jxb/erac477)
Supplement: erac477_suppl_Supplementary_Figures_S1-S11_Table_S1 [file erac477_suppl_supplementary_figures_s1-s11_table_s1.pdf]

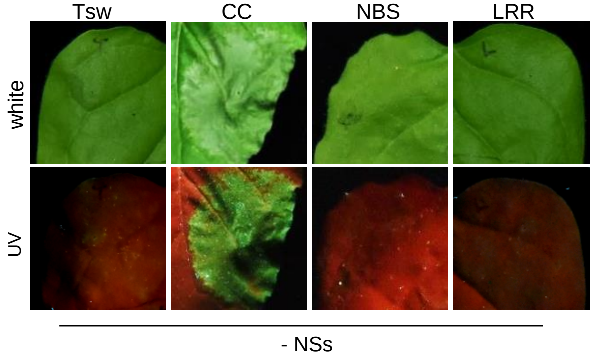

Supplementary Fig. S1 Cell death phenotypes of codon-optimized synthetic *Tsw* (*Tsw-GFP*) or domain truncations (*CC-GFP*, *NBS-GFP*, and *LRR-GFP*) expressed *N. benthamiana* leaves. Photographs were taken 3 days (for domain truncations) or 7 days (for full-length Tsw) post-inoculation (dpi). Images under white (upper) and UV light (lower) are shown.

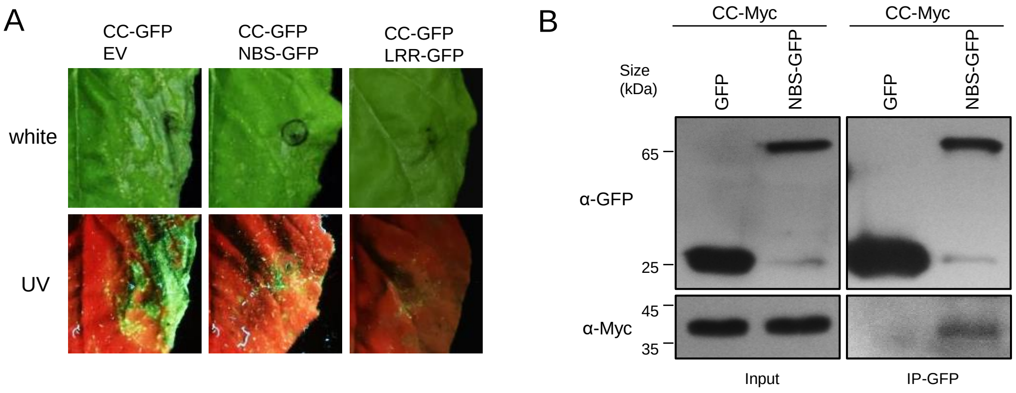

Supplementary Fig. S2 Both NBS and LRR domains of Tsw attenuated the autoactivation activity of CC domain. (A) NBS-GFP and LRR-GFP were co-expressed with CC-GFP in *N. benthamiana* leaves respectively. The cell death phenotypes were photographed three days post infiltration. The empty vector (GFP) was used as a negative control. (B) Epitope tagged NBS proteins (NBS-GFP) and empty vector (GFP) were transiently co-expressed with CC-Myc in *N. benthamiana*. Three days post infiltration, samples were collected for co-immunoprecipitation analysis. Tagged proteins were enriched with GFP beads, and detected with anti-Myc (α-Myc) and anti-GFP (α-GFP) antibodies.

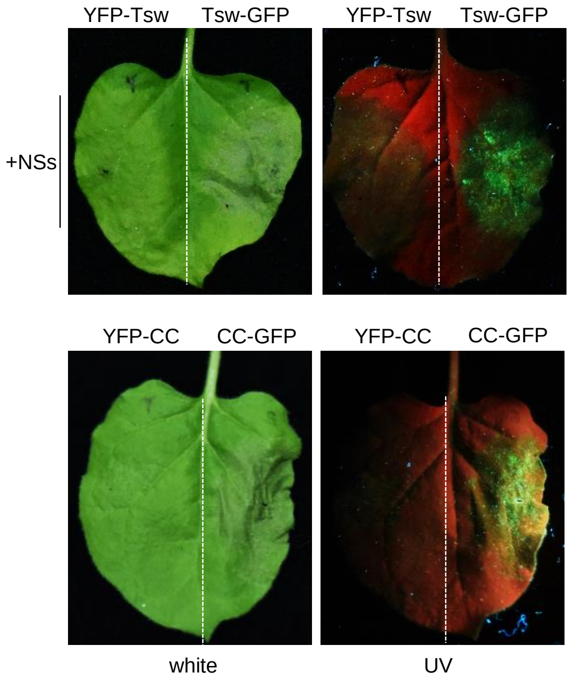

Supplementary Fig. S3 N-terminal free of fusion is critical for cell death induction of Tsw. Epitope tag was fused at the N- or C-terminus of Tsw and Tsw CC domain respectively. The fused protein were expressed individually (YFP-CC, CC-GFP) or together with NSs (YFP-Tsw, Tsw-GFP) in *N. benthamiana*. Cell death phenotypes were photographed at 3 days- (CC domain) or 7 days- (full length NLR) post inoculation (dpi) under white or UV light.

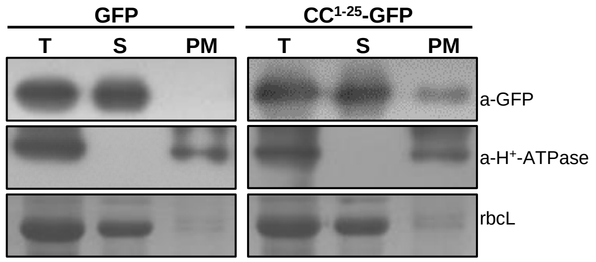

Supplementary Fig. S4 CC^{1-25} fragment re-localize cytoplasmic GFP protein onto the PM. The N-terminal fragment of CC domain (1-25 aa) was fused to GFP protein for membrane fractionation assay. Total protein (T) of GFP and CC^{1-25}-GFP expressed in *N. benthamiana* leaf cells were extracted and separated into soluble (S) and plasma membrane (PM) fractions. The PM-localized H^+-ATPase was used as a positive control.

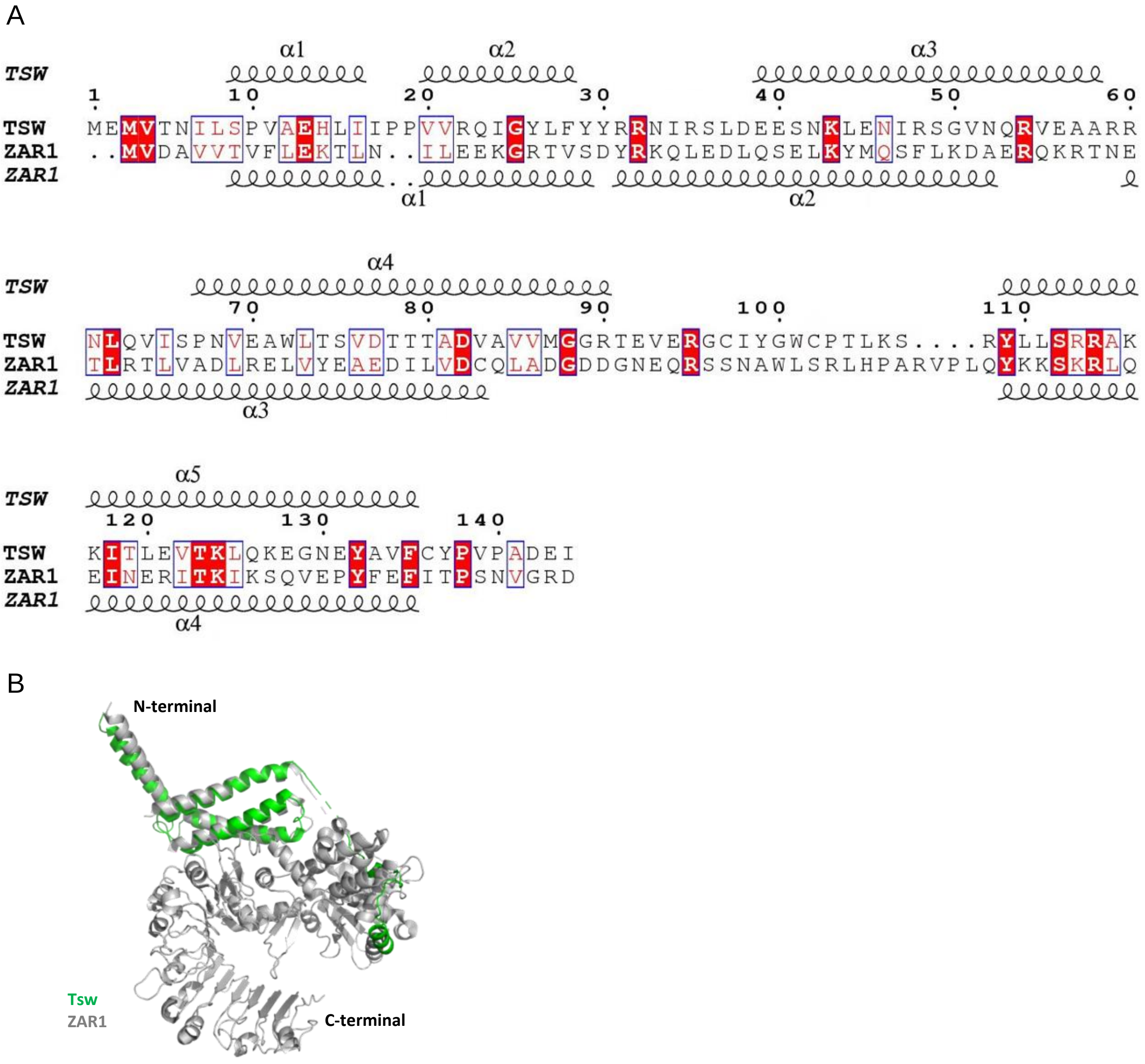

Supplementary Fig. S5 Structural-based sequence alignment of Tsw CC and ZAR1 CC. (A) The amino acid sequence alignment of Tsw CC and ZAR1 CC. (B) Predicted structure model of Tsw CC domain with the template ZAR1 (PDB ID: 6J5T). The green structure indicates the Tsw CC domain and the gray one indicates the ZAR1.

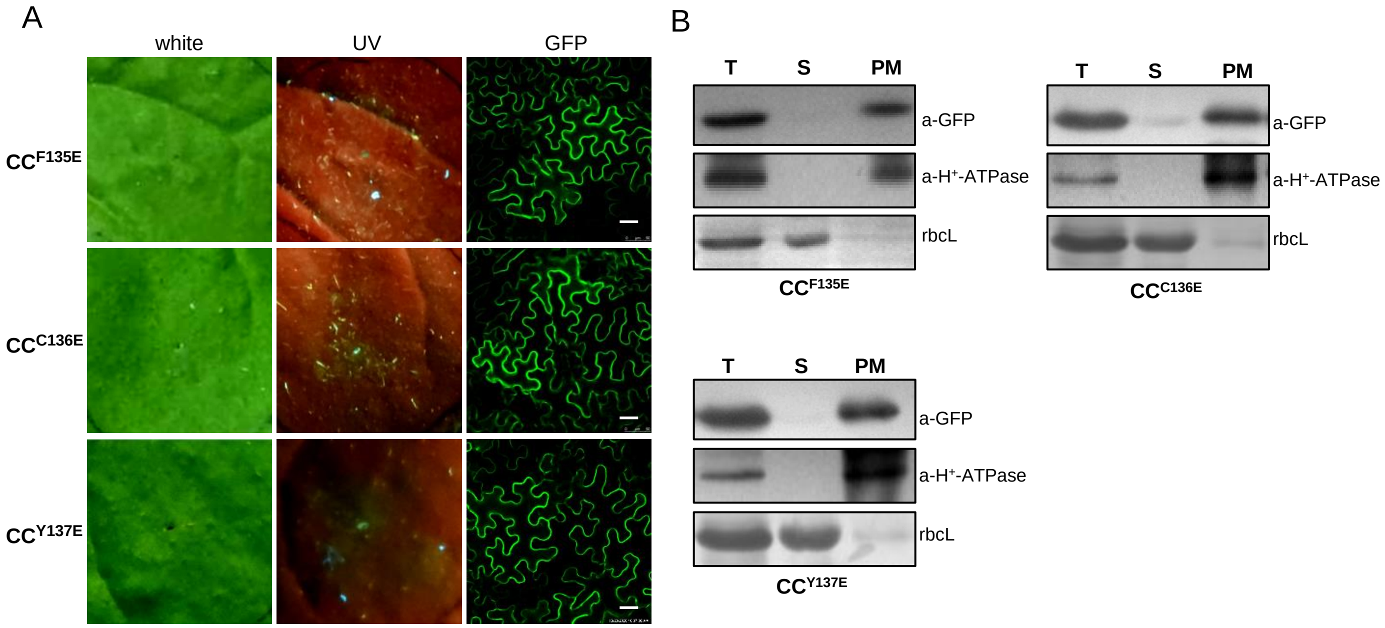

Supplementary Fig. S6 Amino acids of the hydrophobic groove in Tsw CC domain are critical for its autoactivation. (A) Point mutations of each 135th-137th amino acid in CC domain were transiently expressed in *N. benthamiana* leaves individually. The cell death phenotypes and protein localization of each CC mutants were visualized three days post infiltration. (B) Total protein (T) was separated into soluble (S) and plasma membrane (PM) fractions. GFP and the plasma membrane marker H+-ATPase antibodies were used.

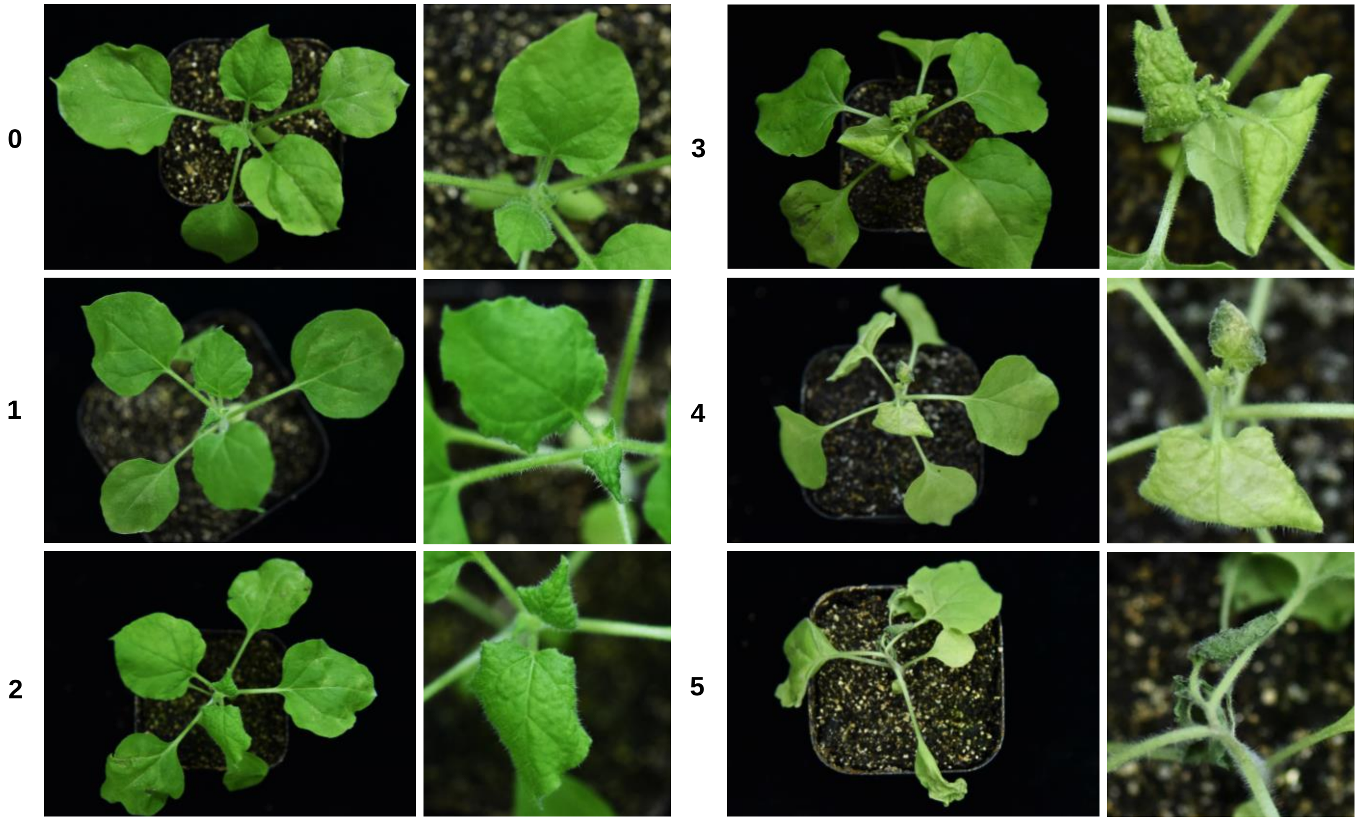

Supplementary Fig. S7 TSWV-infection caused symptoms scale. Representative examples of disease symptoms on *N. benthamiana* scored to 0-5 used to calculate disease index were shown. 0, no disease symptoms; 5, totally death.

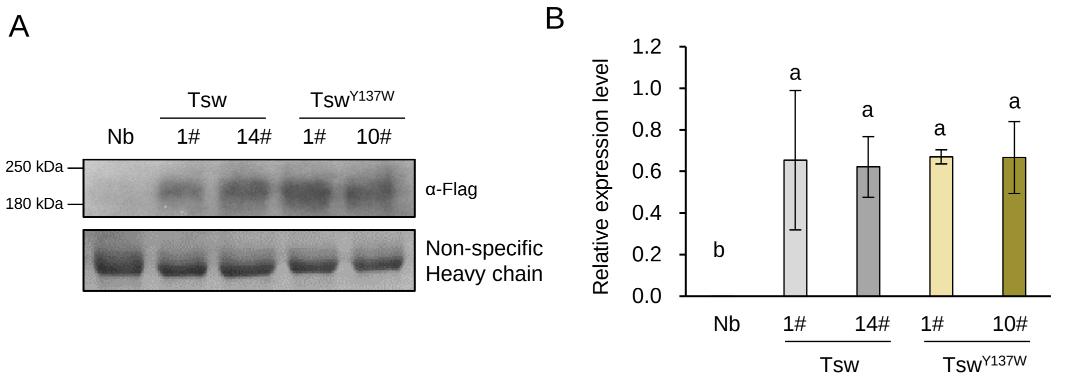

Supplementary Fig. S8 Detection of *Tsw* and *TSW*^{Y137W} transgenic *N. benthamiana* plants. (A) Western blot identify the protein expression of TSW and TSW^{Y137W} in transgenic plants. The protein were enriched by immunoprecipitation with flag beads. Antibodies against Flag were used. The non-specific bands of heavy chain in antiserum were used as loading control. (B) RT-qPCR detects the mRNA level among different transgenic lines. Values are means ± SD, n=6, $P < 0.05$, ANOVA, followed by Duncan's multiple range test.

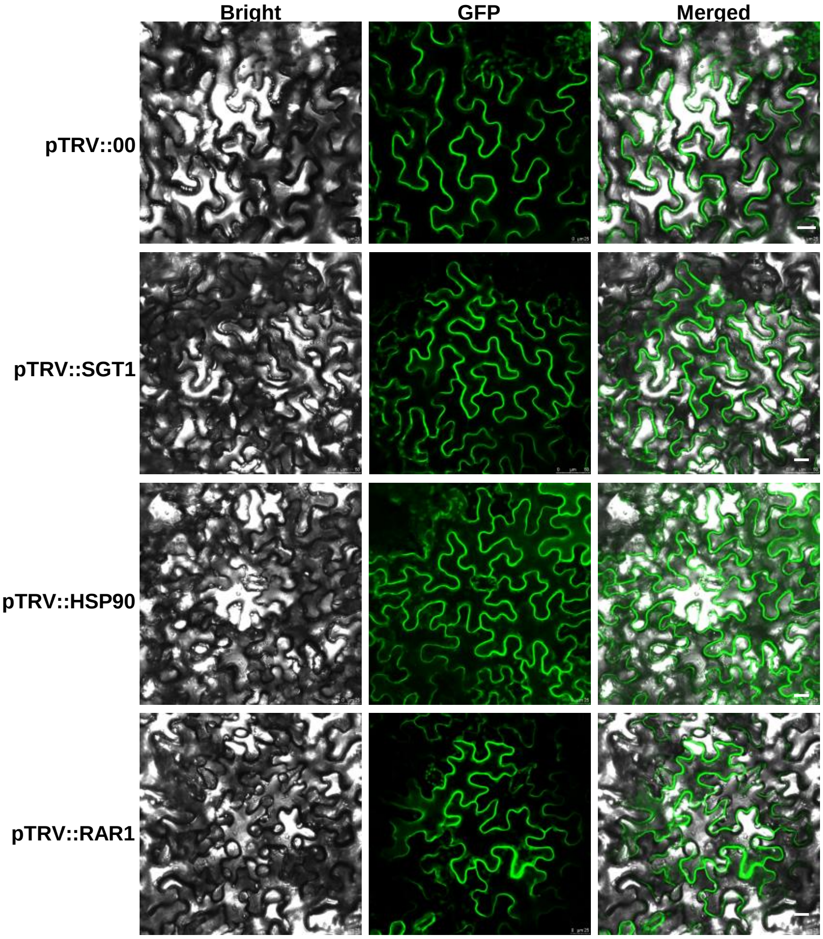

Supplementary Fig. S9 Tsw CC domain is localized to the PM in *SGT1*-, *RAR1*-, and *HSP90*-silenced *N. benthamiana* plants. Subcellular localization of CC-GFP in pTRV::00, pTRV::SGT1, pTRV::RAR1, and pTRV::HSP90 constructs expressed *N. benthamiana* leaves were shown. Bars = 50 µm.

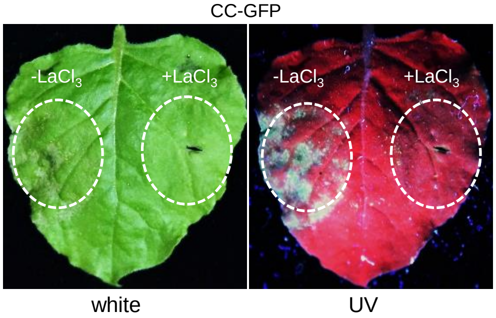

Supplementary Fig. S10 Tsw CC mediated cell death is Ca^{2+} dependent. CC-GFP was transiently pre-expressed in *N. benthamiana* leaves for 12 h, then followed by 5 mM $LaCl_3$ infiltration. The phenotype of leaves was photographed under UV and white light 3-days post inoculation.

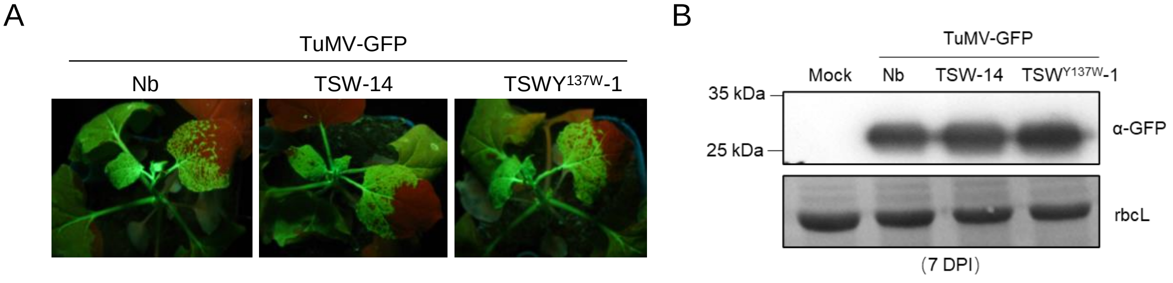

Supplementary Fig. S11 Effect of *Tsw* transgenic plants on TuMV-GFP infection. (A) *Agrobacterium* harbors TuMV-GFP was agroinfiltrated into Nb, Tsw, and Tsw^{Y137W} transgenic *N. benthamiana* lines. The GFP fluorescence of systemically infected leaves was photographed under long-wave UV light at 7 dpi. (B) Western blot detection of GFP accumulation in systemically infected leaves of figure S11A. rbcL was used as loading control.

Table S1: DNA primers used in this study.

Gene	Sequence (5'-3')
CC-Tsw-F(GFP)	CTACAAGATCTCGAGATGGAAATGGTAACTAATATTTTG
CC-Tsw-R(GFP)	CCCGTCGACAAGCTTGAAAGCTGTCATGACCTCTT
m-cc-1R (Y137W)	TGGAACCGGCCAGCAGAAAACAG
m-cc-2F (Y137W)	CTGTTTTCTGCTGGCCGGTTCCA
m-cc-1R (Y137S)	TGGAACCGGAGAGCAGAAAACAG
m-cc-2F (Y137S)	CTGTTTTCTGCTCTCCGGTTCCA
m-cc-1R (Y137E)	TGGAACCGGTTCGCAGAAAACAG
m-cc-2F (Y137E)	CTGTTTTCTGCGAACCGGTTCCA
m-cc-1R (Y137F)	TGGAACCGGAAAGCAGAAAACAG
m-cc-2F (Y137F)	CTGTTTTCTGCTTTCCGGTTCCA
m-cc-1R (Y137A)	TGGAACCGGAGCGCAGAAAACAG
m-cc-2F (Y137A)	CTGTTTTCTGCGCTCCGGTTCCA
m-cc-1R (F135E)	TGGAACCGGATAGCACTCAACAG
m-cc-2F (F135E)	CTGTTGAGTGCTATCCGGTTCCA
m-cc-1R (F135W)	CGGATAGCACCAAACAGCATACTC
m-cc-2F (F135W)	GAGTATGCTGTTTGGTGCTATCCG
m-cc-1R (C136E)	TGGAACCGGATACTCGAAAACAG
m-cc-2F (C136E)	CTGTTTTCGAGTATCCGGTTCCA
m-cc-1R (V134E)	ATAGCAGAATTCAGCATACTCGTT
m-cc-2F (V134E)	AACGAGTATGCTGAATTCTGCTAT
m-cc-1R (V134W)	ATAGCAGAAAACCCAATACTCGTT
m-cc-1R (V134W)	AACGAGTATGCTTGGTTCTGCTAT
1-135aa-CC-R	CCCGTCGACAAGCTTGAAAACAGCATACTCGTTAC
1-136aa-CC-R	CCCGTCGACAAGCTTGCAGAAAACAGCATACTCGT
1-137aa-CC-R	CCCGTCGACAAGCTTATAGCAGAAAACAGCATACT
30-169aa-CC-F	CTACAAGATCTCGAGATGAGGCGCAACATCAGGTCTC
41-169aa-CC-F	CTACAAGATCTCGAGATGTCTAATAAGCTCGAGAATA
CC-Tsw-F (Myc)	CAAGCTCGAGATGGAAATGGTAACTAATATTTTG
CC-Tsw-R (Myc)	CAAGGTCGACAAAGCTGTCATGACCTCTT
NBS-Tsw-F	CAAGGGTACCATGAAAGATGAGGAGATCACTATTGT
NBS-Tsw-R	CAAGCTCGAGTTATGCTCACCCTCAGAC
LRR-Tsw-F	CTACAAGATCTCGAG ATGTTTTTGGTGAACCACAACGT
LRR-Tsw-R	AGATCCGGTGGATCCAGACCTACCGTCTGACATAG
VIGS-HSP90-F	TGAGTAAGGTTACCGAATTC ACAGGATGCTGAAGCTTGGG
VIGS-HSP90-R	AGACGCGTGAGCTCGGTACC AGAGCCAATAAATTATACCC
VIGS-RAR1-F	TGAGTAAGGTTACCGAATTC CATGAAGCTATTATTACGGA
VIGS-RAR1-R	AGACGCGTGAGCTCGGTACC GTACAAACACAGCGAAATAT
VIGS-SGT1-F	TGAGTAAGGTTACCGAATTC CCTGTATGAAGCTTGAAGAG
VIGS-SGT1-R	AGACGCGTGAGCTCGGTACC ATCCGTATGTCTCAATTTCG
Nb-EF1α-F	TGGTGTCCTCAAGCCTGGTATGGTTG
Nb-EF1α-R	ACGCTTGAGATCCTTAACCGCAACATTCTT
QNb-HSP900-F-1	CGAAATCACGAAGGAAGGGT
QNb-HSP900-R-1	TTGGGCTTCTTCCTTGTGTC
QNb-RAR1-F	GCCAGAGGATCGGTTGCA
QNb-RAR1-R	TGCAGGAGTTTTCAGGGTTATCA
QNb-SGT1-F	GCCGATGAGGACACCAGAA

QNb-SGT1-R	GACAGAACAGTCCCATTAGATTCCA
SlANL-1-F	CTACAAGATCTCGAG ATGGAATTCTTCTCATATGG
SlANL-1-R	CCCGTCGACAAGCTT CTCTTGCAATTTTCTAGAAT
SlANL-1-R (Y135E)	CTCGCAGAAAACAGCATAAT
SlANL-1-2F (Y135E)	ATTATGCTGTTTTCTGCGAG
SlANL-1-1R (Y135W)	CCAGCAGAAAACAGCATAAT
SlANL-1-2F (Y135W)	ATTATGCTGTTTTCTGCTGG
